# MARCKS Protein Is Phosphorylated and Regulates Calcium Mobilization during Human Acrosomal Exocytosis

**DOI:** 10.1371/journal.pone.0064551

**Published:** 2013-05-21

**Authors:** Marcelo J. Rodriguez Peña, Jimena V. Castillo Bennett, Osvaldo M. Soler, Luis S. Mayorga, Marcela A. Michaut

**Affiliations:** 1 Laboratorio de Biología Reproductiva, Universidad Nacional de Cuyo, Mendoza, Argentina; 2 Laboratorio de Biología Celular y Molecular, Instituto de Histología y Embriología, Facultad de Ciencias Médicas, Universidad Nacional de Cuyo, Mendoza, Argentina; 3 Instituto de Ciencias Básicas, Universidad Nacional de Cuyo, Mendoza, Argentina; BioScience Project, United States of America

## Abstract

Acrosomal exocytosis is a calcium-regulated exocytosis that can be triggered by PKC activators. The involvement of PKC in acrosomal exocytosis has not been fully elucidated, and it is unknown if MARCKS, the major substrate for PKC, participates in this exocytosis. Here, we report that MARCKS is expressed in human spermatozoa and localizes to the sperm head and the tail. Calcium- and phorbol ester-triggered acrosomal exocytosis in permeabilized sperm was abrogated by different anti-MARCKS antibodies raised against two different domains, indicating that the protein participates in acrosomal exocytosis. Interestingly, an anti-phosphorylated MARCKS antibody was not able to inhibit secretion. Similar results were obtained using recombinant proteins and phospho-mutants of MARCKS effector domain (ED), indicating that phosphorylation regulates MARCKS function in acrosomal exocytosis. It is known that unphosphorylated MARCKS sequesters PIP_2_. This phospholipid is the precursor for IP_3_, which in turn triggers release of calcium from the acrosome during acrosomal exocytosis. We found that PIP_2_ and adenophostin, a potent IP_3_-receptor agonist, rescued MARCKS inhibition in permeabilized sperm, suggesting that MARCKS inhibits acrosomal exocytosis by sequestering PIP_2_ and, indirectly, MARCKS regulates the intracellular calcium mobilization. In non-permeabilized sperm, a permeable peptide of MARCKS ED also inhibited acrosomal exocytosis when stimulated by a natural agonist such as progesterone, and pharmacological inducers such as calcium ionophore and phorbol ester. The preincubation of human sperm with the permeable MARCKS ED abolished the increase in calcium levels caused by progesterone, demonstrating that MARCKS regulates calcium mobilization. In addition, the phosphorylation of MARCKS increased during acrosomal exocytosis stimulated by the same activators. Altogether, these results show that MARCKS is a negative modulator of the acrosomal exocytosis, probably by sequestering PIP_2_, and that it is phosphorylated during acrosomal exocytosis.

## Introduction

The acrosomal exocytosis is a calcium-regulated secretion required for physiological fertilization in mammalian sperm [1]. During this synchronized and tightly regulated process, the outer membrane of the acrosome and the plasma membrane fuse at multiple sites in the anterior region of the sperm head and, as a result, the acrosome content is released. Exocytosis of the sperm acrosome –also called acrosome reaction – happens once in the lifespan of the spermatozoa and is a prerequisite for sperm fusion with the egg membrane.

Protein kinase C (PKC) has been proposed as a key component of the acrosomal exocytosis transduction pathway; however, PKC role in the process is not completely understood [2]. It has been documented that phorbol esters, known PKC activators, trigger acrosome exocytosis in different species [3]–[6], and that levels of DAG, a natural agonist of PKC, increase when human sperm are stimulated by progesterone or the calcium ionophore A23187 [7]. The mechanism of PKC participation in acrosomal exocytosis is still unclear and much less is known about its potential targets. Myristoylated alanine-rich C kinase substrate (MARCKS) is known as the major cellular substrate for PKC in many cell types. MARCKS has been implicated in the regulation of brain development and postnatal survival, cellular migration and adhesion, as well as phagocytosis, endocytosis, and exocytosis [8]. The presence of MARCKS has been described previously in rat testis by Western blot [9]. In this work, authors showed by immunohistochemical studies that MARCKS appears equally during all stages of spermatogenesis, except in mature spermatozoa [9]. Because of this, the expression of MARCKS in mature sperm is unclear.

MARCKS contains three highly conserved domains. The first domain at the amino terminus contains the consensus sequence for myristoylation and is responsible, together with the third domain, for association of MARCKS with lipid membranes. The second domain, also known as MH2 domain, resembles the cytoplasmic tail of the cation-independent mannose-6-phosphate receptor but its function is unknown. The third domain is referred to as the effector domain (ED) or phosphorylation site domain and contains four serine residues that can be phosphorylated by PKC. When not phosphorylated, this domain can bind calmodulin with high affinity, crosslink microfilaments of actin *in vitro* [10], [11] or bind to PIP_2_ [12]. ED phosphorylation results in loss of these activities, and frequently is accompanied by translocation from the membrane to the cytosol [8].

The aim of this study was to determine the expression of MARCKS in human sperm and to describe its participation in acrosomal exocytosis using two different and complementary models: permeabilized and non-permeabilized sperm. Our results show that MARCKS is present in human sperm and localizes to the sperm head and the tail. In permeabilized cells, we report that MARCKS has an inhibitory role in stimulated acrosomal exocytosis and that this inhibitory function is abolished by phosphorylation. In living sperm, using a permeable peptide corresponding to MARCKS ED, we show that MARCKS also inhibits exocytosis and, in addition, abrogates calcium mobilization induced by progesterone. We also demonstrated that the level of phosphorylated MARCKS increases during acrosomal exocytosis activation by a natural inducer, such as progesterone, and pharmacological activators, such as calcium ionophore and phorbol ester. Our results show that MARCKS is a negative modulator in acrosomal exocytosis, which is phosphorylated during acrosomal exocytosis, and regulates calcium mobilization.

## Materials and Methods

### Ethics Statement

Human semen samples were obtained from normal healthy donors. The participants provided their written informed consent to participate in this study. The informed consent and protocol for semen handling were carried out in accordance with the Declaration of Helsinki (2008) and approved by the Ethic Committee of the School of Medicine, Universidad Nacional de Cuyo.

### Reagents

Recombinant streptolysin O (SLO) was obtained from Dr. Bhakdi (University of Mainz, Mainz, Germany). Protein kinase C βII (PKC βII), Adenophostin A, 2-aminoethoxydiphenyl borate (2-APB) and progesterone from Calbiochem were purchased from Merck Química Argentina S.A.I.C. (Buenos Aires, Argentina). Phosphatidylinositol 4, 5-bisphosphate (PIP_2_) was from Avanti Polar Lipids, Inc., (Alabaster, USA). Prestained molecular weight markers were from Bio Rad (Buenos Aires, Argentina). A goat polyclonal anti-MARCKS antibody that recognizes the N-terminus of human MARCKS (anti-MARCKS N-19) and blocking peptide MARCKS N-19 were from Santa Cruz Biotechnology, Inc. (Santa Cruz, CA). A mouse monoclonal anti-MARCKS that recognizes the N-terminus corresponding to amino acids 2–66 of human MARCKS (anti-MARCKS) and a rabbit polyclonal anti-MARCKS that recognizes a peptide around the serine 162 of MARCKS effector domain (anti-MARCKS ED) were from Abcam. Rabbit polyclonal anti-phosphorylated MARCKS (pSer^152/156^, anti-phospho-MARCKS) antibody, anti-β-tubulin antibody, phorbol 12-myristate 13-acetate (PMA) and fluorescein isothiocyanate-coupled *Pisum sativum* agglutinin (FITC-PSA) were obtained from Sigma-Aldrich (Buenos Aires, Argentina). A23187 was from Alomone Laboratories Ltd. (Jerusalem, Israel). Cy3-labeled goat anti-rabbit antibody and DyLight488™-labeled donkey anti-mouse antibody were from Jackson Immunochemicals (Sero-immuno Diagnostics, Inc. Tucker, GA). Streptavidin-HRP and biotinylated secondary antibodies were purchased from DAKO, Tecnolab (Buenos Aires, Argentina). Tetramethylrhodamine isothiocyanate- labeled *Lens culinaris* agglutinin (TRITC-LCA) was from Vector Labs (Bioars, Argentina). Tetramethylrhodamine-labeled human MARCKS peptide (ED-TMR) was from Anaspec (USA). Glutathion-sepharose was from GE Healthcare (Buenos Aires, Argentina). All other chemicals were purchased from Sigma-Aldrich or Tecnolab (Buenos Aires, Argentina).

### Site-directed mutagenesis of MARCKS effector domain

Plasmid encoding the effector domain of mouse MARCKS, pGE3X-MARCKS (residues 96–184) fused to GST (wt MARCKS ED) was kindly provided by Dr. Jae-Won Soh (University of Inha, Incheon, Korea). MARCKS effector domain (ED) mutants were prepared from pGEX-MARCKS plasmid using the Phusion site-directed mutagenesis kit (New England Bio Labs). MARCKS ED mutant carrying a Ser-to-Ala (MARCKS ED4A) or Ser-to-Asp (MARCKS ED4D) substitutions at amino acid positions 152, 156, 159 and 163 were constructed by conventional PCR using the following 5′ *in vitro* phosphorylated mutagenic oligonucleotides: 5′-GAAGCGCTTT

GCCTTCAAGAAGGCCTTCAAGCTGGCCGGCTTCGCCTTCAAGAAGAGC -3′ (MARCKS ED4A primer; forward) and 5′-GCGCTTTGACTTCAAGAAGGACTTC

AAGCTGGACGGCTTCGACTTCAAGCCG-3′ (MARCKS ED4D primer; forward). The reverse primer was 5′-TTTTTTTTTTTCGGGGTCTCGCTGCTGGGCGACGG-3′. Ligation, transformation and analysis of transformants were performed according to manufactureŕs instructions. Mutations were confirmed by sequencing.

### Recombinant proteins

Plasmids encoding wild type MARCKS ED, the non-phosphorylable MARCKS mutant (MARCKS ED4A), and the phosphomimetic MARCKS mutant (MARCKS ED4D) were transformed in BL21 *E. coli* cells (Stratagene, La Jolla, CA, USA) and expression was induced 3 h at 37°C with 0.5 mM isopropyl 1-thio-D-galactopyranoside. Recombinant proteins were purified by affinity chromatography on glutathione–sepharose beads following standard procedures.

### Acrosomal exocytosis assays

Acrosomal exocytosis assays were performed as previously described [13]. Briefly, human semen obtained from normal healthy donors was allowed to liquefy for 30-60 min at 37°C. We used a swim-up protocol to isolate highly motile sperm under capacitating conditions in Human Tubal Fluid media (HTF, as formulated by Irvine Scientific, Santa Ana, CA) supplemented with 0.5% bovine serum albumin (BSA) for 1 h at 37°C in an atmosphere of 5% CO_2_/95% air. Sperm concentrations were adjusted to 5–10×10^6^ cells/ml and incubated for at least 2 h under capacitating conditions. We have shown previously that human sperm incubated under these conditions are responsive to progesterone [14]. The use of these incubation times permits a direct comparison with our previous published results in permeabilized [13], [15]–[18] and non-permeabilized sperm [19], [20]. Spermatozoa were used without permeabilization (living human sperm) or permeabilized. In permeabilized sperm assays, spermatozoa were resuspended in cold phosphate buffered saline (PBS) containing 2.1 U/ml SLO for 15 min at 4°C. Cells were washed once with PBS and resuspended in ice-cold sucrose buffer (250 mM sucrose, 0.5 mM EGTA, 20 mM Hepes-K, pH 7.4) containing 2 mM dithiothreitol. For all acrosome exocytosis assays, we added inhibitors and stimulants sequentially as indicated in the figures, and incubated for 10–15 min at 37°C after each addition. Sperm were spotted on teflon-printed slides, air dried, and fixed/permeabilized in ice-cold methanol for 1 min. Acrosomal status was evaluated by staining with FITC-coupled *Pisum sativum* agglutinin (FITC-PSA) according to [21]. At least 200 cells were scored using a Nikon Optiphot II microscope equipped with epifluorescence optics. Basal (no stimulation, “control”) and positive (0.5 mM CaCl_2_, corresponding to 10 µM free calcium, “Ca^2+^”) controls were included in all experiments. For each experiment, acrosomal exocytosis indexes were calculated by subtracting the number of reacted spermatozoa in the negative control (range 10%–30%) from all values, and expressing the resulting values as a percentage of the acrosome exocytosis observed in the positive control (range 25%–40%). The average difference between positive and negative controls was 12% (experiments where the difference was less than 10% were discarded). Data were evaluated using one-way ANOVA and post hoc tests (Dunnett's test or confident intervals). Differences were considered significant at the p<0.05 level.

### Preparation and addition of lipids to sperm

Lipids stocks were prepared and added as described previously [22]. Briefly, 1 M PMA stock in DMSO was prepared and a N_2_ stream was applied before storing at −20°C.

We added 0.5 µl of the stock to 49.5 µl of DMSO to obtain a 0.01 M solution. From this stock we prepared additional dilutions in sucrose buffer until getting a 10 µM PMA solution. One microliter of the 10 µM stock was added to 49 µl of sperm suspension to get a final concentration of 200 nM. Phosphatidylinositol–4, 5–bisphosphate (PIP_2_) was dissolved in chloroform∶ methanol∶ water (20∶9∶1, v/v), vortexed and evaporated. The lipid was then dissolved in chloroform and dried under a nitrogen stream. PIP_2_ micelles were made by suspending the lipid in sucrose buffer at a final concentration of 2.5 mM (stock solution), followed by several minutes of sonication at maximum power. One microliter of the 2.5 mM solution was added to 49 µl of sperm suspension in sucrose buffer to get a final concentration of 50 µM PIP_2_.

### SDS-PAGE and Western blots

To examine the presence of MARCKS and phosphorylated MARCKS, the fraction of motile sperm capacitated for 2–3 h in BSA supplemented-HTF medium was used. After removing the HTF by washing cells twice with cold PBS, sperm pellets were resuspended in sample buffer (2% SDS, 10% glycerol, and 62.5 mM Tris-HCl pH 6.8) without disulfide reducing agents. Proteins were extracted by heating three times to 95°C for 4 min each. Extracts were centrifuged (12,000×*g*) for 10 min, and the supernatants were adjusted to 5% β-mercaptoethanol. Protein were resolved on 10% polyacrylamide gels according to [23], and transferred to PVDF membranes (Millipore). Non-specific reactivity was blocked by incubation for 1 h at room temperature (RT) with 5% skim milk dissolved in tris-buffered saline with 0.1% Tween-20 (T-TBS: 50 mM Tris-HCl pH 7.4, 150 mM NaCl, 0.1% Tween-20). Blots were incubated with primary anti-MARCKS antibody from Abcam (0.26 µg/ml), anti-MARCKS N-19 from Santa Cruz (0.13 µg/ml), or anti-phospho-MARCKS (0.06 µg/ml), in blocking solution overnight at 4°C. Biotinylated, goat anti-mouse-IgG (0.076 µg/ml), biotinylated, rabbit anti-goat-IgG (0.4 µg/ml) or biotinylated, goat anti-rabbit-IgG (0.076 µg/ml) were used as secondary antibodies during 60 min incubations at RT. Blots were then incubated with peroxidase-conjugated streptavidin (0.17 µg/ml) during 60 min incubations at RT. Excess first and second antibodies, and peroxidase-conjugated streptavidin were removed by washing five times for 10 min each in T-TBS. For loading control blots were reprobed with anti-β-tubulin. Detection was accomplished with an enhanced chemiluminescence system (ECL; GE Healthcare) and visualized with a Fujifilm LAS-4000 Scanner (Fujifilm, Tokyo, Japan).

### *In vitro* phosphorylation assays

15 µM MARCKS ED domains were incubated in 20 mM HEPES-K pH 7.4, 2 mM DTT, 100 µM ATP, 5 mM MgCl_2_, containing 0.6 U/ml of PKC βII, 140 µM phosphatidylserine, 325 nM PMA, and 100 µM CaCl_2_ for 40 min at 37°C. After incubation, the mixtures were filtered through a Sephadex G-25 spin column equilibrated with sucrose buffer to eliminate PMA and small molecules. Filtered mixtures were kept at −70°C until use. For ^32^P-labelling of the recombinant proteins, 5 µM MARCKS ED, MARCKS ED4A, MARCKS ED4D, and GST were incubated with PKC βII as described above with the addition of protease inhibitors (0.6 mM PMSF and 1 mM leupeptin). The ATP concentration was reduced to 10 µM containing 5 µCi/ml [γ^32^P] ATP (NEN PerkinElmer, Migliore Laclaustra, Argentina). Proteins were resolved in 10% Tris-glycine SDS-PAGE gels. The gels were dried and exposed to Pierce CL-XPosure Film (Tecnolab, Argentina).

### Indirect immunofluorescence

Capacitated sperm were adjusted to 7 ×10^6^ cells/ml, spotted on poly-L-lysine-coated slides, and fixed in 2% paraformaldehyde in PBS for 10 min at RT. After fixation, sperm were incubated in 50 mM glycine–PBS for 10 min at RT and permeabilized with 0.1% Triton X-100 for 10 min. Non-specific reactivity was blocked for 1 h in 5% BSA in PBS. Cells were labeled with anti-MARCKS antibody (overnight at 4°C, 135 nM in 2% BSA) followed by a DyLight488-labeled anti-mouse IgG (1 h at RT, 3 µg/ml in PBS containing polyvinylpyrrolidone, PBS/PVP) or anti-phospho-MARCKS (overnight at 4°C, 135 nM in 2% BSA) followed by a Cy3-labeled anti-rabbit IgG (1 h at RT, 3 µg/ml in PBS/PVP). In some experiments, the anti-phospho-MARCKS antibody was preincubated with phosphorylated MARCKS-ED domain (1.35 µM). Cover slips were washed with 0.1% PVP in PBS between incubations. Finally, the cells were incubated 1 min in cold methanol, and stained with FITC-PSA or TRITC-LCA for 40 min at RT, and washed with PBS/PVP. Cover slips were mounted in 1% Vectashield (Vector Labs). Sperm cells were analyzed by fluorescence microscopy using an Olympus Confocal FV1000 and processed with the program FV10-ASW1.7.

### Intracellular calcium measurements of human spermatozoa

Calcium levels were measured in sperm suspensions using the intracellular fluorescent probe Fluo-3-AM. Motile sperm were adjusted to a concentration of 5–10 ×10^6^ cells/ml and loaded with the permeable form of the dye (Fluo-3-AM, 2 µM) for 30 min at 37°C. Cells were washed once and resuspended in HTF. Sperm suspensions were transferred to thermostated (37°C) cuvettes for fluorescence measurements and stirred constantly. At the indicated times, 15 µM progesterone was added to the samples. When indicated, sperm where incubated 30 min in presence of 4 µM of the permeable tetramethylrhodamine-labeled MARCKS ED (ED-TMR) before adding progesterone. Fluo-3 fluorescence (λ_Ex_ = 505 nm, λ_Em_ = 525 nm emission) was recorded in an AMINCO-Bowman Series 2 (AB2) spectrofluorometer. Data were collected during 600 seconds at a frequency of 0.5 Hz. To calibrate the maximal response, intracellular calcium concentration ([Ca^2+^]*_i_*) was determined using Triton X-100 (0.1%). Measurements were performed at least three times with different batches of sperm. The criterion we used for inclusion of the results obtained with a given sample into the statistical analysis was the normal response to 15 µM progesterone.

## Results and Discussion

### MARCKS is expressed in human spermatozoa

The acrosomal exocytosis is a regulated exocytosis in which the outer membrane of the acrosome and the plasma membrane fuse in the anterior region of the sperm head. Fusion at multiple sites between these membranes causes the release of the acrosomal contents and the loss of the membranes surrounding the acrosome. This particular exocytosis is a synchronized and tightly regulated process, with no recycling of membranes. In the last few years, we have shown that acrosomal exocytosis is mediated by a molecular mechanism that is homologous to that reported for secretion in neuroendocrinal cells [24].

**Figure 1:**
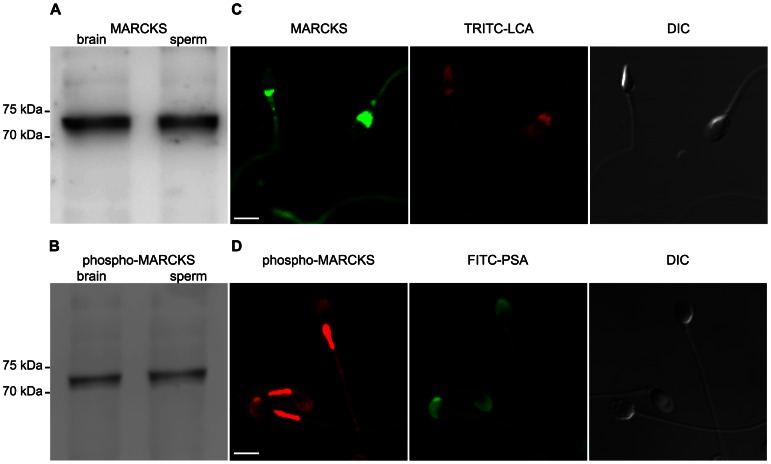
MARCKS is expressed in human sperm. (**A**) Proteins from whole sperm homogenates (sperm, 5×10^6^ cells) were resolved in 10% SDS-PAGE gels and immunoblotted with the anti-MARCKS antibody raised against the N-terminus of MARCKS. Brain extracts (brain) was used as a positive control. (**B**) Identical to A but immunoblotted with the anti-phospho-MARCKS antibody. (**C**) Sperm were double-stained with the anti-MARCKS antibody followed by an anti-mouse-DyLight488-conjugated antibody and with tetramethylrhodamine isothiocyanate-labeled *Lens culinaris* agglutinin to stain acrosomes (TRITC-LCA). (**D**) Sperm were double-stained with the anti-phospho-MARCKS antibody followed by an anti-rabbit-Cy3 antibody and FITC-coupled *Pisum sativum* agglutinin to stain acrosomes (FITC-PSA). Shown are representative images from three different experiments. DIC, cells observed with differential interference contrast. Bars = 5 µm

MARCKS has been implicated in neurosecretion and exocytosis [25]–[28]; nevertheless the precise molecular mechanism of MARCKS in exocytosis is not well known and it is unknown if it participates in the signal transduction pathway of acrosomal exocytosis. The presence of MARCKS in human sperm is still unclear. Mosevitsky and Silicheva have reported that MARCKS is present in all stages of spermatogenesis in rat testis; however, they did not find MARCKS in mature spermatozoa [9], [29]. This prompted us, first, to determine the presence of MARCKS in human sperm by Western blot. The theoretical molecular mass of MARCKS is 32 kDa; however on SDS-PAGE, MARCKS shows an anomalous migration corresponding to a 80 kDa protein due to its non-globular, elongated forms and the weak binding of SDS molecules to the highly acidic amino acids [8]. Proteins from mouse brain and whole human sperm extracts were resolved by SDS-PAGE, transferred to PVDF membranes and probed with antibodies raised against either the N-terminal domain (anti-MARCKS and anti-MARCKS N-19, see Materials and Methods for more details) or the effector domain of phosphorylated MARCKS (anti-phospho-MARCKS). Using the anti-MARCKS antibody, immunoblot analysis of whole cell extracts from human sperm demonstrated the presence of a single protein band comigrating with mouse brain MARCKS. The apparent molecular mass of MARCKS was 73 kDa, suggesting that a related, if not identical, form is present in these cells (Fig. 1A). Similar results were obtained when a different antibody raised against the N-terminal domain of MARCKS (anti-MARCKS N-19) was assayed (Fig. S1A). Preincubation with an excess of a blocking peptide corresponding to the N-terminal domain of MARCKS reduced the signal substantially, indicating that antibodies were specific (Fig. S1A). Human sperm samples were also probed with a polyclonal antibody raised against a synthetic phosphorylated peptide derived from the region of rat MARCKS that is phosphorylated on serines 152 and 156 (anti-phospho-MARCKS antibody). As shown in Fig. 1B, the antibody recognized a single band of the expected molecular weight (73 kDa).

Next, using the same antibodies, indirect immunostaining was used to localize MARCKS on fixed sperm. MARCKS localized to the head and the tail of human sperm. The N-terminal MARCKS antibodies showed a punctuate pattern in acrosomal region and a homogenous pattern in the postacrosomal region in 98% of acrosome intact cells (Fig. 1C and S1B). It is important to point out that this punctate pattern of MARCKS has been previously described in other cells such as neurons [30]. The phospho-MARCKS antibody showed a uniform pattern in the acrosomal region without staining in the postacrosomal region of the sperm head in 89% of acrosome intact cells (Fig. 1D and S1C). All antibodies showed a uniform labeling in the tail of all cells evaluated. The preincubation of anti-phospho-MARCKS antibody with an *in vitro* phosphorylated recombinant MARCKS peptide abolished the signal of phospho-MARCKS in the acrosomal region and the tail, indicating that staining was specific. Blocking experiments also showed that label on the midpiece of the flagellum was unspecific (Fig. S1C). Altogether, these results indicate that both phosphorylated and non-phosphorylated MARCKS proteins are present in capacitated human spermatozoa and localize to the head and the tail.

### MARCKS participates in acrosomal exocytosis in human sperm

To investigate whether MARCKS plays a role in acrosomal exocytosis in human sperm, we attempted to inhibit the function of the endogenous protein by treating permeabilized cells with different antibodies raised against main domains involved in MARCKS function: the N-terminal domain and the effector domain.

**Figure 2:**
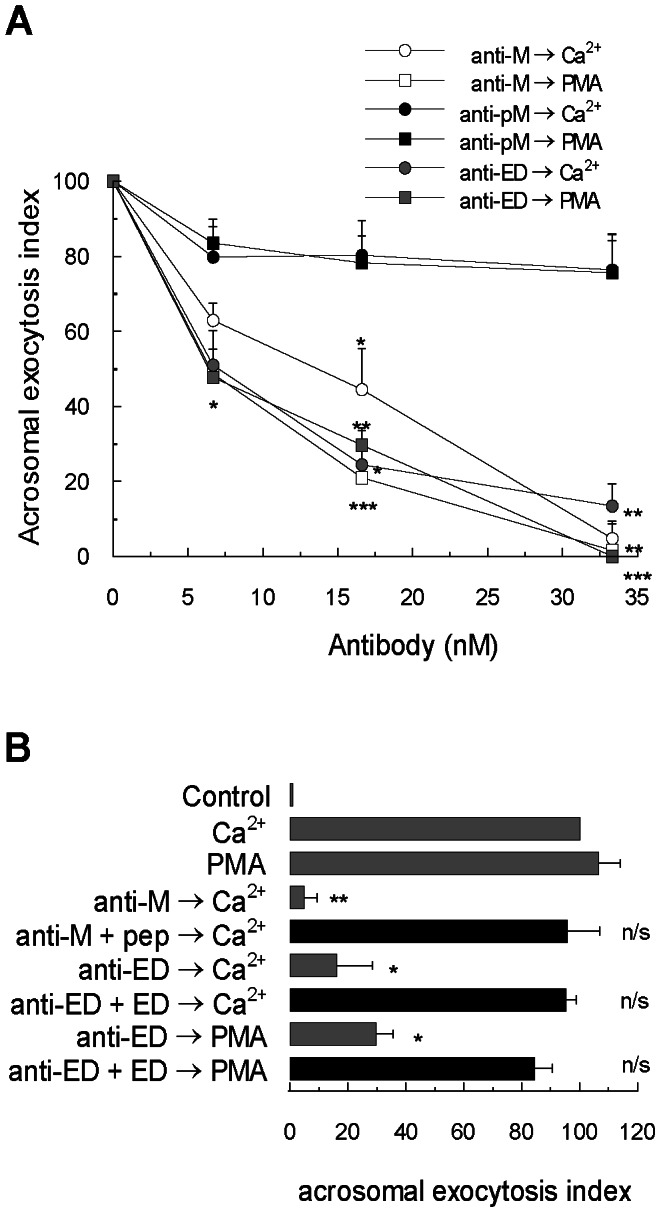
MARCKS participates in acrosomal exocytosis. (**A**) Permeabilized sperm were incubated for 15 minutes at 37°C in the presence of increasing concentrations of anti-MARCKS N-19 antibody (anti-M, white symbols), anti-MARCKS ED (anti-ED, gray symbols) and anti-phospho-MARCKS (anti-pM, black symbols). Acrosomal exocytosis was initiated by adding 10 µM free Ca^2+^ (circles) or 200 nM PMA (squares). For all conditions, the incubation continued for an additional 15 minutes and acrosomes were evaluated by lectin binding. (**B**) Permeabilized sperm were incubated for 15 minutes at 37°C in the presence of 33 nM anti-MARCKS N-19 antibody (anti-M) preincubated with an excess (1∶ 10) of blocking peptide MARCKS N-19 (anti-M+pep) and in the presence of 33 nM anti-MARCKS ED antibody (anti-ED) preincubated with an excess (1∶10) of MARCKS effector domain (anti-ED+ED). Acrosomal exocytosis was then initiated by adding 10 µM free Ca^2+^ or 200 nM PMA and the incubation continued for an additional 15 minutes (black bar). Control experimental conditions (gray bars) include background acrosomal exocytosis in the absence of any stimulation (control), acrosomal exocytosis stimulated by 10 µM free Ca^2+^ (Ca^2+^) and by 200 nM PMA (PMA) and the effect of antibodies without peptide preincubation anti-MARCKS N-19 (anti-M) and anti-MARCKS ED (anti-ED). The percentage of reacted sperm was normalized as described in Materials and Methods. The data represent the means±SEM of at least three independent experiments. The asterisks indicate significant differences from similar conditions stimulated with Ca^2+^ (**, p<0.01; *, p< 0.05); n/s, not significant difference.

We first tested antibodies against the N-terminal domain. Permeabilized sperm were incubated with increasing concentrations of the antibody raised against the N-terminus, anti-MARCKS N-19 antibody, prior to addition of calcium or phorbol 12-myristate 13-acetate (PMA), a permeable analog of DAG. As shown in Fig. 2A (white symbols), the antibody abrogated the acrosomal exocytosis stimulated by both activators. When stimulated with calcium, the half maximal inhibitory concentration (IC_50_, mean±SEM) for anti-MARCKS N-19 antibody was 15±3 nM. When stimulated with PMA, the IC_50_ was 7±2 nM. The inhibitory effect of the anti-MARCKS N-19 antibody was blocked when the antibody was preincubated with the blocking peptide N-19 (Fig. 2B). Similar results were obtained when the other anti-MARCKS antibody raised against the N-terminal domain, anti-MARCKS, was assayed (data not shown). The addition of a non-immune goat antibody had no effect (data not shown). These results indicate that the inhibitory effect of the antibodies was specific. In conclusion, we found that preincubation of cells with antibodies against the N-terminal domain of MARCKS blocks acrosomal exocytosis. Little is known about the function of the N-terminal domain of MARCKS and its participation in membrane fusion. Singer and collaborators have reported that MARCKS is attached to membranes of intracellular mucin granules and that this association is inhibited by a peptide corresponding to the MARCKS N-terminal domain [31]. Takashi and collaborators published that the same peptide inhibited degranulation of human leukocytes *in vitro* [32]. Nevertheless, the mechanism of the N-terminal domain of MARCKS in these secretory processes is not fully understood.

MARCKS effector domain is considered the responsible domain for most of the function of MARCKS, however the mechanism is still unclear [8]. To investigate the participation of MARCKS effector domain in acrosomal exocytosis, we assayed an antibody raised against this domain (anti-MARCKS ED). Permeabilized sperm were incubated with increasing concentrations of this antibody prior to addition of calcium or PMA. As shown in Fig. 2A (gray symbols), the antibody abolished the acrosomal exocytosis stimulated by both activators. When stimulated with calcium, the IC_50_ for anti-MARCKS ED antibody was 7±3 nM. When stimulated with PMA, the IC_50_ was 7±2 nM. The inhibitory effect of the anti-MARCKS ED antibody was blocked when the antibody was preincubated with the recombinant MARCKS ED peptide (Fig. 2B). The addition of a non-immune rabbit antibody showed no effect (data not shown). These results indicate that the inhibitory effect of the anti-MARCK ED antibody might be the result of binding to the effector domain of endogenous MARCKS, perturbing its function in acrosomal exocytosis.

Finally, we assayed increasing concentrations of the anti-phospho-MARCKS antibody in the calcium- and PMA-triggered acrosomal exocytosis. Surprisingly, this antibody had not effect on stimulated exocytosis and concentrations up to 33 nM of anti-phospho-MARCKS antibody showed no inhibition (Fig. 2A, black symbols). Prior studies indicate that MARCKS phosphorylation abrogates membrane binding of MARCKS in many cell types, because neutralization of the positive charges of the basic residues by the phosphoserine residues abolishes the electrostatic contribution of the MARCKS effector domain to membrane binding [8]. Therefore, phosphorylated MARCKS is considered the inactive form of MARCKS [33]. The finding that a phospho-MARCKS antibody was unable to inhibit acrosomal exocytosis is consistent with the idea that phosphorylated MARCKS is inactive in our model when human sperm are stimulated by calcium or PMA.

So far, our results show that MARCKS participates in acrosomal exocytosis, and this participation may be mediated by the N-terminal domain and the non-phosphorylated effector domain.

### The inhibitory role of MARCKS is abolished by phosphorylation

**Figure 3:**
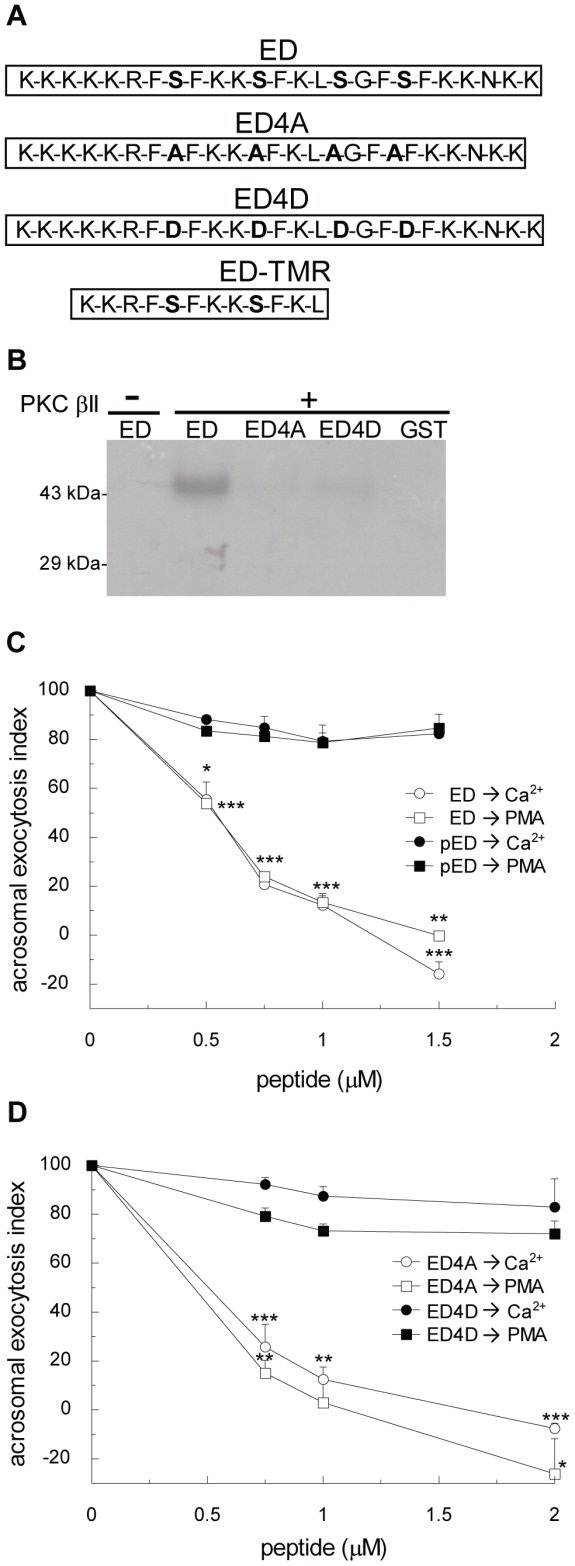
MARCKS inhibits acrosomal exocytosis and this effect is dependent on phosphorylation. (**A**) Amino acid sequence (residues 145–169) of mouse wild type MARCKS effector domain (ED), the non-phosphorylable MARCKS ED mutant (ED4A), the phosphomimetic MARCKS ED mutant (ED4D), and permeable tetramethylrhodamine-conjugated MARCKS ED peptide (ED-TMR). (**B**) Purified GST fusion proteins of wild type MARCKS ED (ED), MARCKS ED4A mutant (ED4A), MARCKS ED4D (ED4D), and GST (26 kDa) were incubated for 40 minutes at 37°C with (+) or without (-) PKC βII under activating conditions in the presence of [γ^32P^]ATP. Samples were then resolved by 10% SDS-PAGE gels, and radiolabeled proteins were detected by autoradiography. Shown is a representative gel from three independent experiments. (**C**) Permeabilized sperm were treated for 15 minutes at 37°C with increasing concentrations of MARCKS effector domain (ED, white symbols) or *in vitro* phosphorylated MARCKS ED (pED, black symbols). Acrosomal exocytosis was initiated by adding 10 µM free Ca^2+^ (circles) or 200 nM PMA (squares). In all conditions, the incubation continued for an additional 15 minutes and acrosomal exocytosis was evaluated by lectin binding. (**D**) Permeabilized sperm were treated for 15 minutes at 37°C with increasing concentrations of MARCKS ED4A (ED4A, white symbols) or MARCKS ED4D (ED4D, black symbols). Acrosomal exocytosis was initiated by adding 10 µM free Ca^2+^ (circles) or 200 nM PMA (squares). In all conditions the incubation continued for an additional 15 minutes and acrosomal exocytosis was evaluated by lectin binding. In C and D, the percentage of reacted sperm was normalized as described in Materials and Methods. The data represent the means±SEM of at least three independent experiments. The asterisks indicate significant differences from similar conditions stimulated with Ca^2+^ (*, p<0.05; **, p<0.01; ***, p<0.001).

MARCKS functions in exocytosis are associated mainly with the MARCKS effector domain. Studies in a variety of isolated or cultured cell types indicate that the phosphorylation state of MARCKS effector domain controls its function. Our results showed that anti-phospho-MARCKS antibody did not affect acrosomal exocytosis whereas the anti-MARCKS ED antibody abolished secretion (Fig. 2). These results suggested that phosphorylated MARCKS ED does not interact with the endogenous machinery in acrosomal exocytosis and raised the question whether non-phosphorylated MARCKS ED does. To test this hypothesis wild type (wt) MARCKS ED was expressed in bacteria as GST fusion protein. After purification, wild type MARCKS ED was incubated under phosphorylating conditions before challenging the acrosome exocytosis (see Materials and Methods and Fig. 3B). Both, non-phosphorylated MARCKS ED and *in vitro* phosphorylated MARCKS ED were tested in the permeabilized sperm acrosomal exocytosis assay stimulated by calcium and PMA. Non-phosphorylated MARCKS ED was able to abrogate secretion (Fig. 3C; IC_50_, 0.55±0.05 µM). On the contrary, phosphorylated MARCKS ED did not abolish stimulated acrosomal exocytosis (Fig. 3C). To eliminate the possibility that the observed results were the consequence of a differential diffusion into permeabilized sperm of phosphorylated and non-phosphorylated MARCKS ED peptide, indirect immunofluorescence against GST was performed. Both, phospho- and non-phosphorylated peptides diffused equally into permeabilized cells (Figure S2). Our results indicated that only non-phosphorylated MARCKS ED interacts with the fusion machinery operating during acrosomal exocytosis.

To further confirm these results, we generated two different mutants of MARCKS ED: the constitutively non-phosphorylable and the constitutively phosphorylated MARCKS (or phosphomimetic) by replacement the 4 serines in the ED with alanine (MARCKS ED4A) and aspartic acid (MARCKS ED4D), respectively (see mutants scheme in Fig. 3A). These mutants were expressed in bacteria as GST-fusion proteins and purified on glutathione-sepharose columns. When these mutants were assayed in acrosome exocytosis, only the MARCKS ED4A mutant inhibited the exocytosis stimulated by calcium and PMA (Fig. 3D; IC_50_, 0.5±0.07 µM) with similar potency than the recombinant non-phosphorylated peptide (compare Fig. 3C and 3D). None of the mutants incorporated γ^32^P ATP in the presence of PKC βII, indicating that these recombinant proteins cannot be phosphorylated (Fig. 3B). The diffusion of MARCKS mutants into permeabilized sperm was also analyzed by indirect immunofluorescence against GST. Both MARCKS ED4A and MARCKS ED4D mutants diffused similarly into permeabilized sperm (Fig S2).

These results are consistent with observations in others secretory cells. A peptide of the MARCKS ED inhibited the PMA-induced norepinephrine release in permeabilized parotid acinar cells [34], [35]. In bovine luteal cells expressing a mutant MARCKS that cannot be phosphorylated by PKC, prostaglandin F_2α_ failed to stimulate oxytocin exocytosis [36]. Our findings indicate that MARCKS is a component of the signal transduction pathways in acrosomal exocytosis and that its inhibitory role is regulated by phosphorylation.

### The inhibitory role of MARCKS is reverted by PIP_2_ and adenophostin

**Figure 4:**
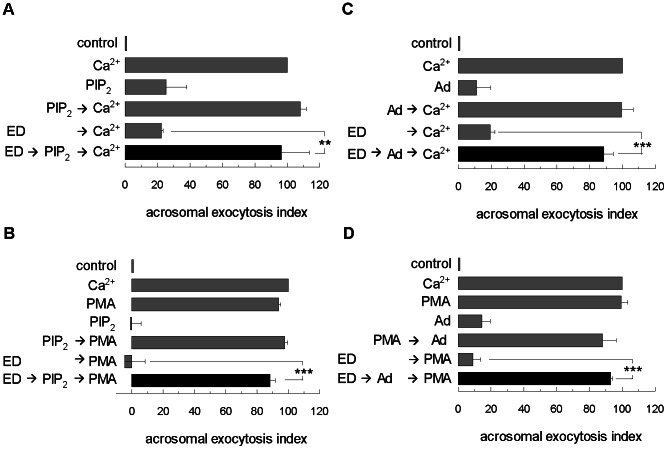
PIP_2_ and adenophostin rescue inhibition of MARCKS effector domain in acrosomal exocytosis. Permeabilized sperm were treated for 15 minutes at 37°C with 1 µM MARCKS effector domain (ED); then, 50 µM PIP_2_ was added for another 15 min and acrosomal exocytosis was initiated by adding 10 µM free Ca^2+^ (**A**) or 200 nM PMA (**B**). The incubation continued for an additional 15 minutes (black bars). Control experimental conditions (gray bars) include background acrosomal exocytosis in the absence of any stimulation (control), acrosomal exocytosis stimulated by 10 µM free Ca^2+^ (Ca^2*+*^) and by 200 nM PMA (PMA), the absence of effect of PIP_2_ with or without acrosomal exocytosis stimulation. Permeabilized sperm were treated for 15 minutes at 37°C with 1 µM MARCKS effector domain (ED); then, 5 µM adenophostin was added (Ad), and acrosomal exocytosis was initiated by adding 10 µM free Ca^2+^ (**C**) or 200 nM PMA (**D**). The incubation continued for an additional 15 minutes (black bars). Control experimental conditions (gray bars) include background acrosomal exocytosis in the absence of any stimulation (control), acrosomal exocytosis stimulated by 10 µM free Ca^2+^ (Ca^2+^) and by 200 nM PMA (PMA), the absence of effect of adenophostin with or without acrosomal exocytosis stimulation, and the inhibitory effect of ED in acrosomal exocytosis. The percentage of reacted sperm was normalized as described in Materials and Methods. The data represent the means±SEM of at least three independent experiments. The asterisks indicate significant differences from similar conditions without PIP_2_ or adenophostin (**, p<0.01; ***, p<0.001).

We found that wt MARCKS ED or mutant MARCKS ED4A inhibited stimulated acrosomal exocytosis and we wondered about the mechanism of the inhibition of these domains. Heo and collaborators [37] found that MARCKS ED binds to the plasma membrane through its association with phosphoinositides, mainly with phosphatidylinositol 4, 5-bisphosphate (PIP_2_) and, because of this, MARCKS has been called pipmodulin [12], [38]. Therefore we proposed that the functional effects we observe in this study are due to PIP_2_ sequestration by wt MARCKS ED or mutant MARCKS ED4A domains. Although regulation of the principal enzymes involved in PIP_2_ synthesis and degradation can contribute to local modulation of PIP_2_ levels in some cases, McLaughlin and collaborators [38] have postulated that synthesis alone is insufficient to maintain lateral concentration gradients of a relatively abundant lipid in the face of diffusion, and that additional mechanisms such as lateral sequestration and local release must also be involved in spatiotemporal control of PIP_2_. Our group has previously proposed that PIP_2_ is continuously produced in a positive loop in human sperm [22]. We hypothesize that MARCKS acts as a pipmodulin in acrosomal exocytosis and that the addition of PIP_2_ would recover acrosomal exocytosis inhibited by MARCKS ED in human sperm. In fact, as shown in Fig. 4, PIP_2_ was able to recover the MARCKS ED inhibition in acrosomal exocytosis stimulated by calcium (Fig. 4A) and by PMA (Fig. 4B).

PIP_2_ generates the second messengers, inositol 1, 4, 5-triphosphate (IP_3_) and diacylglycerol (DAG). An increase on IP_3_ level would activate IP_3_-sensitive calcium channels allowing the efflux of this ion from intracellular stores (most likely the acrosome). Hence, we hypothesize that signaling through MARCKS regulates the intracellular calcium mobilization required for the acrosomal exocytosis. If this was the case, then, MARCKS ED (Fig. 3C) and MARCKS ED4A (Fig. 3D) would block exocytosis because they prevented calcium mobilization. We tested this possibility by promoting intravesicular calcium release with the IP_3_-sensitive calcium channel agonist adenophostin. The sole addition of adenophostin rescued exocytosis impaired by MARCKS ED (Fig. 4C and 4D), supporting the notion that the end point of the MARCKS pathway is the mobilization of intracellular calcium.

Our results indicate that both PIP_2_ and adenophostin are able to reverse the inhibitory effect of non-phosphorylated MARCKS in stimulated acrosomal exocytosis.

These findings suggest that MARCKS inhibits secretion by sequestering PIP_2_ and preventing the release of calcium through IP3-sensitive calcium channels.

### Assessing MARCKS function on acrosomal exocytosis in non-permeabilized sperm

So far all results were obtained using the SLO-permeabilized human sperm model. Our group has previously showed that polybasic amino acids-containing proteins can penetrate the sperm plasma membrane and thus are valuable tools to study sperm physiology in intact cells [19]. Fortunately, MARCKS ED meets this requirement and it can be assayed in acrosomal exocytosis in non-permeabilized sperm. To investigate the function of MARCKS in intact cells, we analyzed the effect of a permeable peptide corresponding to wt MARCKS ED in living human sperm. We used a commercial MARCKS peptide, the tetramethylrhodamine-conjugated peptide sequence KKRFSFKKSFKL (ED-TMR) derived from amino acid residues 154 –165 of human MARCKS ED, which retains the first two of the four serine residues in the ED (see peptide scheme in Fig. 3A). First, we determined the permeability of ED-TMR in living human sperm (see Fig. S3). To rule out that the translocation of the protein was a fixation artifact, cells were treated with trypsin before fixation. Under this condition, permeable ED-TMR peptide was detected mainly in the head of human sperm (Fig. S3). A similar permeable MARCKS peptide has been used in sea urchin sperm and it was found enriched in the acrosome region [39].

**Figure 5:**
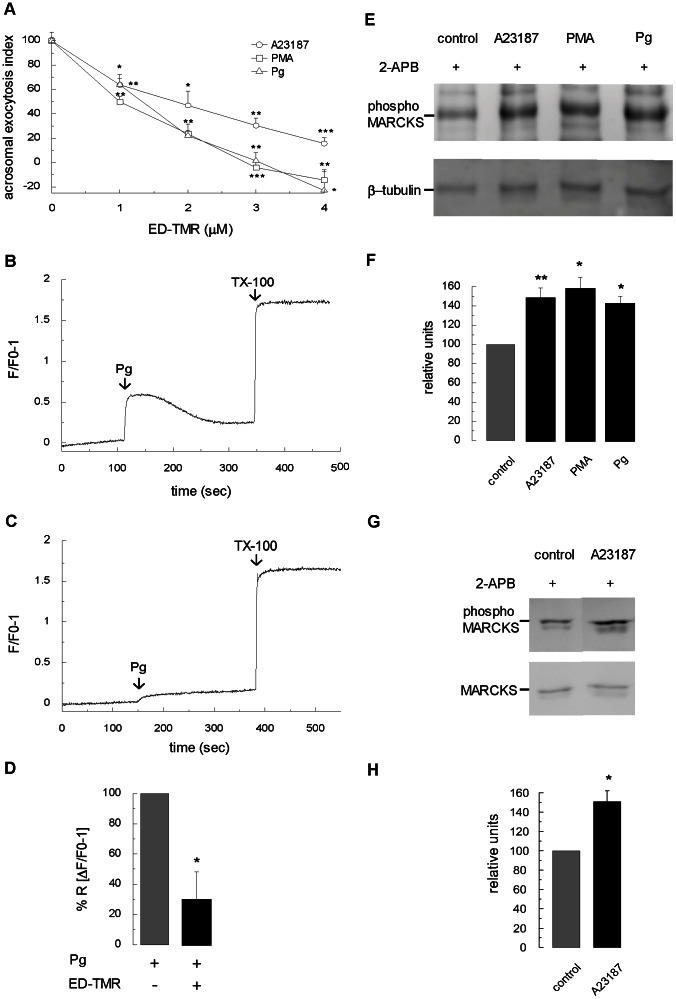
Assessing MARCKS function on acrosomal exocytosis in non-permeabilized sperm. (**A**) Capacitated and non-permeabilized sperm were treated for 30 minutes at 37°C with increasing concentrations of a permeable tetramethylrhodamine-labeled MARCKS ED peptide (ED-TMR). Acrosomal exocytosis was then initiated by adding 10 µM calcium ionophore A23187 (circles), 200 nM PMA (squares) or 15 µM progesterone (triangles), and the incubation continued for 15 minutes. The percentage of reacted sperm was normalized as described in Materials and Methods. The data represent the means±SEM of at least three independent experiments. The asterisks indicate significant differences from similar conditions without ED-TMR (*, p<0.05; **, p<0.01; ***, p<0.001). (**B**) Capacitated and non-permeabilized sperm were loaded with 2 µM Fluo-3-AM and incubated for 30 min at 37°C. At the indicated time (arrow) 15 µM progesterone was added. Maximal [Ca^2+^]*i* response was calibrated with 0.1% Triton X-100 (TX-100, arrow) at the end of the incubation period. Shown are traces representative of three experiments. The increase in fluorescence is expressed as (*F*/*F*0)-1 ((maximum fluorescence intensity/initial fluorescence)-1) *versus* time in seconds. (**C**) Identical to B with previous incubation for 30 min with 4 µM ED-TMR, before adding progesterone (arrow). (**D**) Quantification of three independent experiments. Bars represent mean±SEM; data were normalized to the calcium response to progesterone in the absence of ED-TMR. The asterisk indicates a significant difference from stimulation with progesterone (*, p<0.05; Student's t test). (**E**) Non-permeabilized and capacitated sperm were incubated for 15 min at 37°C with 100 µM 2-APB (control) and then treated for 20 min with 10 µM A23183, 200 nM PMA, or 15 µM progesterone. After treatment, proteins of each condition (5×10^6^ cells) were resolved in a 10% gels and transferred to PVDF membranes. Phosphorylated MARCKS was detected by Western blot assay using an anti-phospho-MARCKS antibody. Anti-β-tubulin antibody was used as loading control. (**F**) Quantification of three independent experiments showed in E. Bars represent mean±SEM; data were normalized against β-tubulin signal in control sperm. The asterisks indicate a significant difference from control sperm (*, p<0.05; **, p<0.01). (**G**) Capacitated and non-permeabilized sperm were incubated for 15 min at 37°C with 100 µM 2-APB (control) and then treated for 20 min with 10 µM A23183. After treatment, proteins of each condition (5×10^6^ cells) were resolved in a 10% gels and transferred to PVDF membranes. Phosphorylated MARCKS was detected by Western blot assay using an anti-phospho-MARCKS antibody. Anti-MARCKS antibody was used as loading control. (**H**) Quantification of three independent experiments showed in G. Bars represent mean±SEM; data were normalized against MARCKS signal in control sperm. Asterisk indicates a significant difference from control sperm (*, p<0.05).

Then, we tested the permeable TMR-labeled peptide in the acrosomal exocytosis assay using non-permeabilized sperm. We found that ED-TMR inhibited the secretion stimulated by calcium ionophore A23187, PMA, and progesterone in a concentration-dependent manner in human sperm (Fig. 5A). Note that the permeable peptide results in intact sperm are similar to those obtained with the recombinant wt MARCKS ED and mutant MARCKS ED4A in permeabilized cells (compare Fig. 5A and Fig. 3C and 3D). It is interesting to note that the acrosomal exocytosis index in the presence of high concentrations of ED-TMR (Fig. 5A), wt MARCKS ED (Fig. 3C), and MARCKS ED4A (Fig. 3D) was significantly lower than the index in unstimulated controls. This indicates that the compounds are inhibiting basal acrosome exocytosis. It has been shown that unphosphorylated MARCKS effector domain crosslinks F-actin in vitro [40]. We think that the addition of MARCKS ED and MARCKS ED4A in permeabilized cells, and ED-TMR in non-permeabilized cells, might be crosslinking F-actin in human sperm and stabilizing the actin cytoskeleton underneath the plasma membrane and, consequently, inhibiting spontaneous acrosome exocytosis. Nevertheless, further studies are needed to elucidate this observation.

To test the hypothesis that MARCKS was involved in calcium mobilization, we measured intracellular calcium levels in human spermatozoa preincubated with the ED-TMR peptide and stimulated with progesterone. Progesterone stimulates a rapid, extracellular calcium-dependent, transient increase in human sperm ([Ca^2+^]*i*) necessary for initiation of the acrosomal exocytosis [41]–[43]. When intact human sperm were preincubated with ED-TMR peptide, the increase in calcium levels caused by progesterone (Fig. 5B) was significantly diminished (Fig. 5C and 5D). Interestingly, these results are similar with those obtained in mast cells, in which a constitutively non-phosphorylable MARCKS mutant inhibited calcium mobilization and degranulation [44]. Our findings validate the hypothesis that MARCKS regulates calcium mobilization in living human sperm.

So far these results show that non-phosphorylated MARCKS inhibited acrosomal exocytosis when stimulated by different activators in both permeabilized and living human sperm. In fact, in SLO-permeabilized spermatozoa, both the recombinant wt MARCKS peptide and a non-phosphorylable MARCKS mutant inhibited exocytosis activated by calcium and PMA (Fig. 3C and 3D, white symbols). In non-permeabilized spermatozoa, a permeable peptide corresponding to the MARCKS effector domain inhibited exocytosis triggered by calcium ionophore A23187, PMA, and progesterone (Fig. 5A). On the contrary, when phosphorylated forms of MARCKS - *in vitro* phosphorylated domain or phosphomimetic MARCKS mutant - were tested in the acrosomal exocytosis assay, they were unable to abrogate exocytosis (Fig. 3C and 3D, black symbols). These results suggest that MARCKS might be inactivated by phosphorylation during acrosomal exocytosis and we tested this hypothesis. We investigated whether MARCKS was phosphorylated during the activation of exocytosis by different stimulators. Human sperm were incubated with 2-aminoethoxy diphenyl borate (2-APB) before stimulation. 2-APB is an IP_3_-sensitive calcium channel inhibitor, which arrests acrosomal exocytosis when outer acrosomal and plasma membranes are in close apposition [45]. Hence, 2-APB avoids the formation of hybrid vesicles preventing the loss of membranes and membrane-associated proteins that would occur during the acrosomal exocytosis. As shown in Fig. 5E and 5F, phosphorylated MARCKS increased significantly when acrosomal exocytosis was stimulated by calcium ionophore A23187, PMA, and progesterone (50%, 60%, and 40%, respectively). These percentages were obtained when data were normalized against ß-tubulin as loading control (Fig. 5F). When data were normalized against MARCKS, similar results were obtained (see Fig. 5G and 5H for the effect of A23187; the results for PMA and progesterone are not shown). These results indicate that the level of phospho-MARCKS increases during acrosomal exocytosis, whereas total MARCKS levels remain constant.

The fact that MARCKS is phosphorylated by different activators during acrosomal exocytosis is compatible with the idea that, in human sperm, non-phosphorylated MARCKS is released from membranes by phosphorylation, increasing the availability of PIP_2_ to generate IP_3_. In addition, PIP_2_ might also interact with proteins involved in membrane fusion such as SNARE and synaptotagmin. A variety of SNARE proteins are expressed in human sperm, including syntaxins 1A, 1B, 4, and 6, SNAP-23 and SNAP-25, and VAMP-2 [46], and studies in other cell types have provided evidence for a role for PIP_2_ in regulating syntaxin-1 and VAMP-2–mediated membrane fusion [47]. Synaptotagmins are a family of Ca^2+^ sensor proteins that participate in multiple exocytotic pathways, including acrosomal exocytosis [15]. Similarly to MARCKS, the binding of synaptotagmin to phospholipids is also regulated by phosphorylation [48].

**Figure 6:**
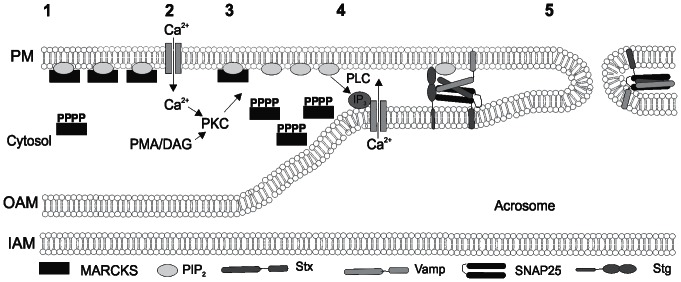
Working model for the role of MARCKS in acrosomal exocytosis. In resting sperm, MARCKS is present in two forms, a phosphorylated form in cytosol and another non-phosphorylated form, which binds to membranes sequestering PIP_2_ (1). When sperm is activated by a natural inducer such as progesterone, acrosomal exocytosis is triggered by a cytoplasmic increase of Ca^2+^ (2). Then, Ca^2+^ and DAG activate conventional PKC. Activated PKC phosphorylates the effector domain of MARCKS. Phosphorylated MARCKS is translocated to cytosol, augmenting the availability of PIP_2_ (3). PIP_2_ is hydrolyzed by PLC to generate IP_3_ and DAG. IP_3_ elicits the efflux of Ca^2+^ from acrosome (4). In addition, PIP_2_ interacts with SNAREs proteins and synaptotagmin (Stg) to fuse plasma and outer acrosomal membranes (5). Stx, syntaxin; VAMP, vesicle associated membrane protein; SNAP-25, synaptosomal-associated protein 25.

In summary, we report the expression of MARCKS in human sperm and its participation in the signal transduction pathway of acrosomal exocytosis. In SLO-permeabilized sperm, we demonstrate that MARCKS inhibits secretion and that this inhibition is regulated by phosphorylation and reverted by PIP_2_ and adenophostin. In living human sperm, we show that a permeable MARCKS peptide abolishes stimulated acrosomal exocytosis and abrogates calcium mobilization stimulated by progesterone. Furthermore, we demonstrate that MARCKS phosphorylation increases during acrosomal exocytosis. Altogether, these results show that MARCKS is a negative modulator in acrosomal exocytosis, probably by sequestering PIP_2_, and that its function is regulated by phosphorylation. On the basis of the results presented here and previous publications from others, we propose a working model for MARCKS function in human sperm (see Fig. 6). In resting sperm, a fraction of MARCKS is phosphorylated and inactive, whereas another fraction is non-phosphorylated and tightly associated to membranes sequestering PIP_2_. When sperm is stimulated, acrosomal exocytosis is triggered by a cytoplasmic increase of Ca^2+^ that –besides several other effects– activates a conventional PKC, which phosphorylate MARCKS effector domain. This phosphorylation would trigger the translocation of phosphorylated MARCKS to cytosol and, as a consequence, would increase the availability of PIP_2_. This lipid can then been hydrolyzed by PLC to generate IP_3_ and DAG. IP_3_ is necessary to elicit the efflux of Ca^2+^ from acrosome, which triggers the final steps of acrosomal exocytosis [49]. Further studies will be required to explore the untested details of this model.

## Supporting Information

Figure S1 **Specificity of anti-MARCKS and anti-phospho-MARCKS antibodies.** (**A**) Postnuclear extract from mouse brain (1 µg de proteins, brain) or capacitated human sperm (5×10^6^ cells, sperm) were resolved in a 10% gels, transferred to PVDF membranes, and probes with two anti-MARCKS antibodies against N-terminal domain, one from Abcam (anti-MARCKS, left panel) and other from Santa Cruz (anti-MARCKS N-19, right panel). In both cases, the primary antibody were preincubated with an excess (1∶10) of a blocking peptide corresponding to the N-terminal of MARCKS (N-19 peptide, Santa Cruz) (ab+pep). The signal reduction for anti-MARCKS antibody was 90% and for anti-MARCKS N-19 was 75%. (**B**) Sperm were double-stained with the anti-MARCKS antibody (Abcam) followed by an anti-mouse-DyLight488 (a and b) or just with the anti-mouse-DyLight488 (c and d). Acrosomes were stained with tetramethylrhodamine isothiocyanate-coupled *L. culinaris* agglutinin (TRITC-LCA, b and d). (**C**) Sperm were double-stained with an anti-phospho-MARCKS followed by an anti-rabbit-Cy3 (a and b), just with the anti-rabbit-Cy3 (c and d) or anti-phospho-MARCKS preincubated with an excess (1∶10) of *in vitro* phosphorylated MARCKS ED domain (phospho-MARCKS+pED, e and f). Acrosomes were stained with FITC-PSA (b, d, and f). Bars =  5 µm
(TIF)

Figure S2 **Recombinant MARCKS ED proteins diffuse equally into permeabilized sperm.** (**A**) Dot blot against GST-fusion proteins: 200 ng purified GST fusion proteins of wild type MARCKS ED (ED), phosphorylated MARCKS ED (pED), MARCKS ED4A mutant (ED4A), MARCKS ED4D (ED4D), and glutathione-S-transferase (GST) were immobilized on PVDF membranes after blocking non-specific reactivity with 5% skim milk dissolved in T-TBS. Membranes were incubated with anti-GST antibody (0.016 µg/ml) from Novus Biologicals (Littleton, CO) in blocking solution overnight at 4°C. A HRP-conjugated goat anti-rabbit-IgG was used as secondary antibody during 1 h at RT (1∶20000 in blocking solution). The experiment was repeated twice with similar results. (**B**) Capacitated and permeabilized sperm were treated for 30 minutes at 37°C with 1 µM of each of the following domains: wild type MARCKS ED (ED), phosphorylated MARCKS ED (pED), MARCKS ED4A mutant (ED4A), and MARCKS ED4D (ED4D). Then, sperm were washed twice with PBS, incubated with an anti-GST antibody (166 nM, 60 min at RT in 3% BSA), washed, and incubated with an anti-rabbit-Cy3 antibody (60 min at RT 3 µg/ml in 1% BSA). After washing, cells were fixed and mounted as described in Materials and Methods. (**C**) Quantification of three independent experiments shown in B. Bars represent mean±SEM. n/s, not significant difference.
(TIF)

Figure S3 **MARCKS peptide permeates into non-permeabilized sperm.** (**A**) Non-permeabilized sperm were treated for 30 minutes at 37°C with 4 µM permeable MARCKS ED domain (ED-TMR) and then incubated with (+) or without (-) 0.5 µg/ml trypsin for 30 minutes at 37°C. Then, cells were fixed and mounted as described in Materials and Methods. (**B**) Quantification of tetramethylrhodamine-labeled acrosome sperm. At least 300 cells were scored. The data represent the means±S.E of at least four independent experiments. DIC, differential interference contrast.
(TIF)
