# Supplementary figures and images for: MARCKS Protein Is Phosphorylated and Regulates Calcium Mobilization during Human Acrosomal Exocytosis
Source: PLoS One. 2013 May 21;8(5):e64551. doi: 10.1371/journal.pone.0064551 (PMC3660367; doi:10.1371/journal.pone.0064551)

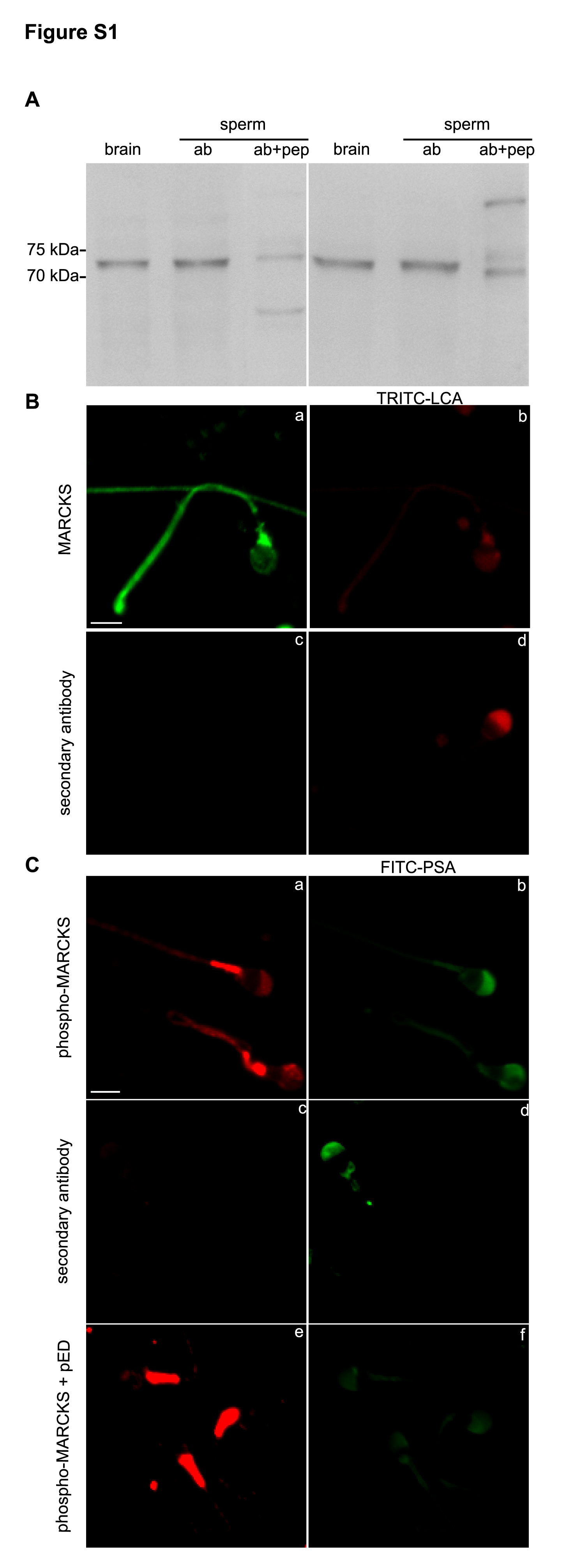

Supplement: Figure S1 — Specificity of anti-MARCKS and anti-phospho-MARCKS antibodies. (A) Postnuclear extract from mouse brain (1 µg de proteins, brain) or capacitated human sperm (5×10^6^ cells, sperm) were resolved in a 10% gels, transferred to PVDF membranes, and probes with two anti-MARCKS antibodies against N-terminal domain, one from Abcam (anti-MARCKS, left panel) and other from Santa Cruz (anti-MARCKS N-19, right panel). In both cases, the primary antibody were preincubated with an excess (1∶10) of a blocking peptide corresponding to the N-terminal of MARCKS (N-19 peptide, Santa Cruz) (ab+pep). The signal reduction for anti-MARCKS antibody was 90% and for anti-MARCKS N-19 was 75%. (B) Sperm were double-stained with the anti-MARCKS antibody (Abcam) followed by an anti-mouse-DyLight488 (a and b) or just with the anti-mouse-DyLight488 (c and d). Acrosomes were stained with tetramethylrhodamine isothiocyanate-coupled L. culinaris agglutinin (TRITC-LCA, b and d). (C) Sperm were double-stained with an anti-phospho-MARCKS followed by an anti-rabbit-Cy3 (a and b), just with the anti-rabbit-Cy3 (c and d) or anti-phospho-MARCKS preincubated with an excess (1∶10) of in vitro phosphorylated MARCKS ED domain (phospho-MARCKS+pED, e and f). Acrosomes were stained with FITC-PSA (b, d, and f). Bars = 5 µm (TIF) [file pone.0064551.s001.tif]

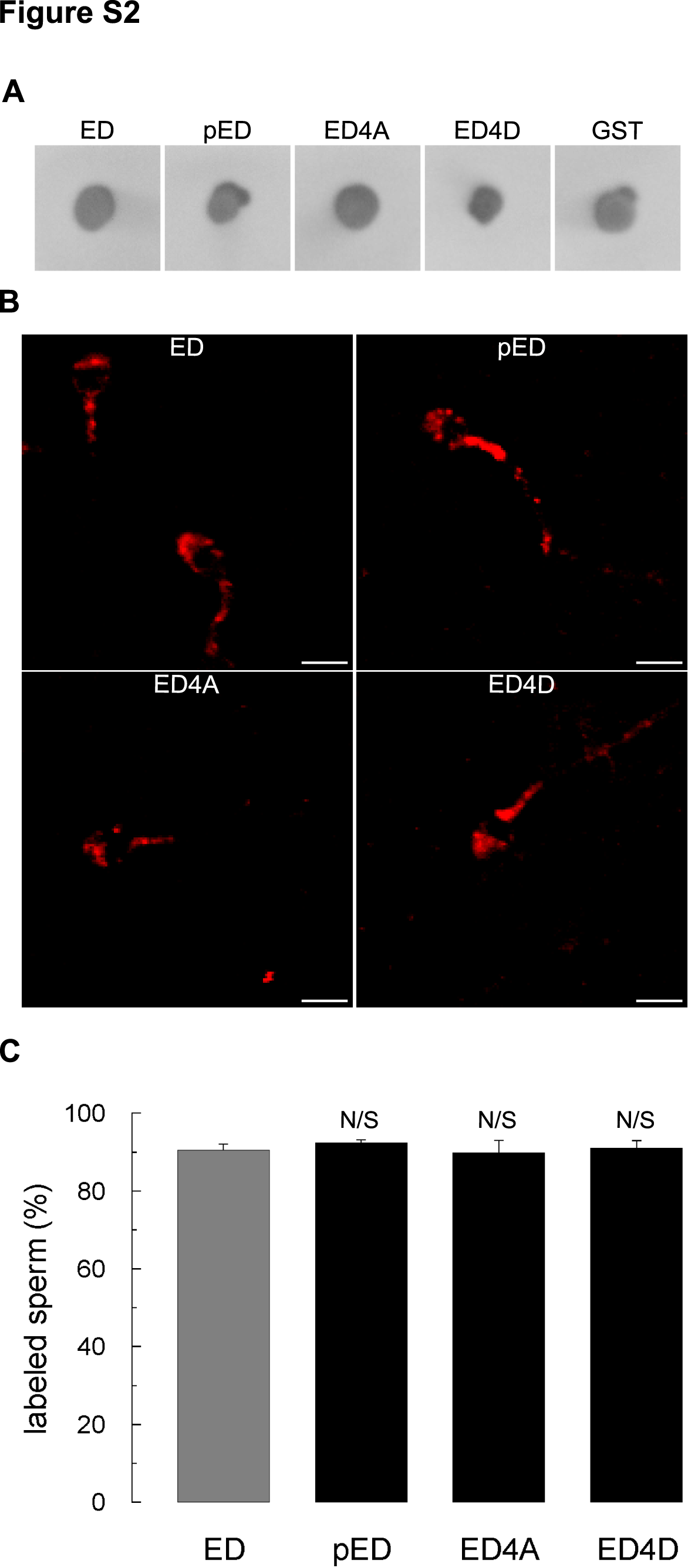

Supplement: Figure S2 — Recombinant MARCKS ED proteins diffuse equally into permeabilized sperm. (A) Dot blot against GST-fusion proteins: 200 ng purified GST fusion proteins of wild type MARCKS ED (ED), phosphorylated MARCKS ED (pED), MARCKS ED4A mutant (ED4A), MARCKS ED4D (ED4D), and glutathione-S-transferase (GST) were immobilized on PVDF membranes after blocking non-specific reactivity with 5% skim milk dissolved in T-TBS. Membranes were incubated with anti-GST antibody (0.016 µg/ml) from Novus Biologicals (Littleton, CO) in blocking solution overnight at 4°C. A HRP-conjugated goat anti-rabbit-IgG was used as secondary antibody during 1 h at RT (1∶20000 in blocking solution). The experiment was repeated twice with similar results. (B) Capacitated and permeabilized sperm were treated for 30 minutes at 37°C with 1 µM of each of the following domains: wild type MARCKS ED (ED), phosphorylated MARCKS ED (pED), MARCKS ED4A mutant (ED4A), and MARCKS ED4D (ED4D). Then, sperm were washed twice with PBS, incubated with an anti-GST antibody (166 nM, 60 min at RT in 3% BSA), washed, and incubated with an anti-rabbit-Cy3 antibody (60 min at RT 3 µg/ml in 1% BSA). After washing, cells were fixed and mounted as described in Materials and Methods. (C) Quantification of three independent experiments shown in B. Bars represent mean±SEM. n/s, not significant difference. (TIF) [file pone.0064551.s002.tif]

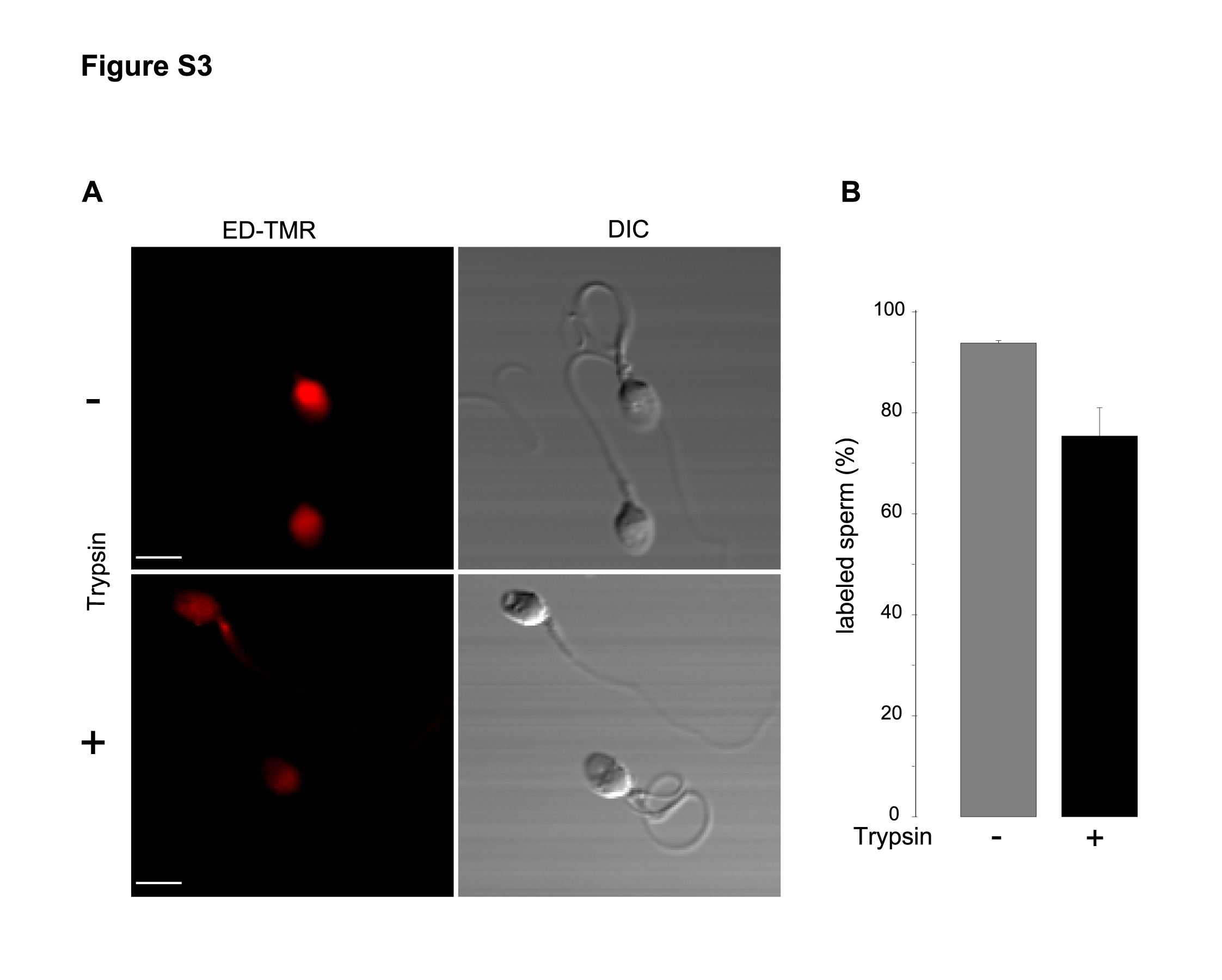

Supplement: Figure S3 — MARCKS peptide permeates into non-permeabilized sperm. (A) Non-permeabilized sperm were treated for 30 minutes at 37°C with 4 µM permeable MARCKS ED domain (ED-TMR) and then incubated with (+) or without (-) 0.5 µg/ml trypsin for 30 minutes at 37°C. Then, cells were fixed and mounted as described in Materials and Methods. (B) Quantification of tetramethylrhodamine-labeled acrosome sperm. At least 300 cells were scored. The data represent the means±S.E of at least four independent experiments. DIC, differential interference contrast. (TIF) [file pone.0064551.s003.tif]
